# A comprehensive review: synergizing stem cell and embryonic development knowledge in mouse and human integrated stem cell-based embryo models

**DOI:** 10.3389/fcell.2024.1386739

**Published:** 2024-04-22

**Authors:** Cathérine Dupont

**Affiliations:** Department of Developmental Biology, Erasmus University Medical Center, Rotterdam, Netherlands

**Keywords:** synthetic embryo models, stem cell-based embryo models, mouse embryogenesis, human embryogenesis, embryonic development, developmental biology, stem cells

## Abstract

Mammalian stem cell-based embryo models have emerged as innovative tools for investigating early embryogenesis in both mice and primates. They not only reduce the need for sacrificing mice but also overcome ethical limitations associated with human embryo research. Furthermore, they provide a platform to address scientific questions that are otherwise challenging to explore *in vivo*. The usefulness of a stem cell-based embryo model depends on its fidelity in replicating development, efficiency and reproducibility; all essential for addressing biological queries in a quantitative manner, enabling statistical analysis. Achieving such fidelity and efficiency requires robust systems that demand extensive optimization efforts. A profound understanding of pre- and post-implantation development, cellular plasticity, lineage specification, and existing models is imperative for making informed decisions in constructing these models. This review aims to highlight essential differences in embryo development and stem cell biology between mice and humans, assess how these variances influence the formation of partially and fully integrated stem cell models, and identify critical challenges in the field.

## Introduction

Headlines featuring “Synthetic Embryo Models” (SEMs) or “Artificial Embryos” are sensational, but can be misleading and potentially cause unnecessary concern among the general public. These terms serve attempts to describe stem cell-based embryo models, which are further categorized into non-integrated and integrated stem cell models (Rossant and Tam, 2021; Hyun et al., 2020). The non-integrated models focus on specific aspects of embryonic development; while the integrated models, such as blastoids and ETX embryoids, simulate the progressive development of the entire mammalian conceptus, including its extra-embryonic tissues. The forthcoming review further classifies integrated stem cell-based embryo models into partially and fully integrated types. Fully integrated models encompass all extra-embryonic lineages, whereas partially integrated models represent only a subset of these extra-embryonic lineages. Integrated stem cell-based embryo models can take shape through an assembly approach, involving the aggregation of various appropriate early lineage-specific stem cells that are known to mutually influence each other’s development. Alternatively, they can also be constructed through an inductive approach, where the formation of the stem cell-based embryo model depends on elaborate cell culture media that will chemically dictate the fate of the used cells. These models are created from biological materials and are thus far from being synthetic or artificial. The primary goal of designing and using stem cell-based embryo models is not to generate human or animal beings from *in vitro* entities. They rather offer a versatile approach to study early mammalian embryonic development and provide valuable insights into cellular processes and molecular mechanisms, all without the need for real human embryos or sacrificing pregnant lab mice. Their versatility enables researchers to assess specific aspects of mammalian embryonic development, making them effective tools for scientific research and advancements in animal and human reproductive medicine. Furthermore, for drug testing and screening, these models provide a controlled environment enabling the assessment of drug efficacy, in supporting complicated pregnancies, as well as evaluating potential embryo toxicity during pregnancy. In the upcoming sections, this review comprehensively addresses the significant differences in embryo development and stem cell biology between mice and humans, highlighting how these variances influence the production methods of partially and fully integrated stem cell-based embryo models tailored to each species. Moreover, it critically examines recent developments in both human and mouse partially and fully integrated stem cell models, offering insights into the notable challenges encountered within this field. Additionally, the review explores the potential of non-human primate embryos and non-human primate stem cell-based embryo models in advancing knowledge of primate embryogenesis, particularly in contexts where ethical limitations surrounding human embryos and human stem cell-based embryo models restrict research.

## Pre- and post-implantation development in mice *versus* humans

**FIGURE 1:**
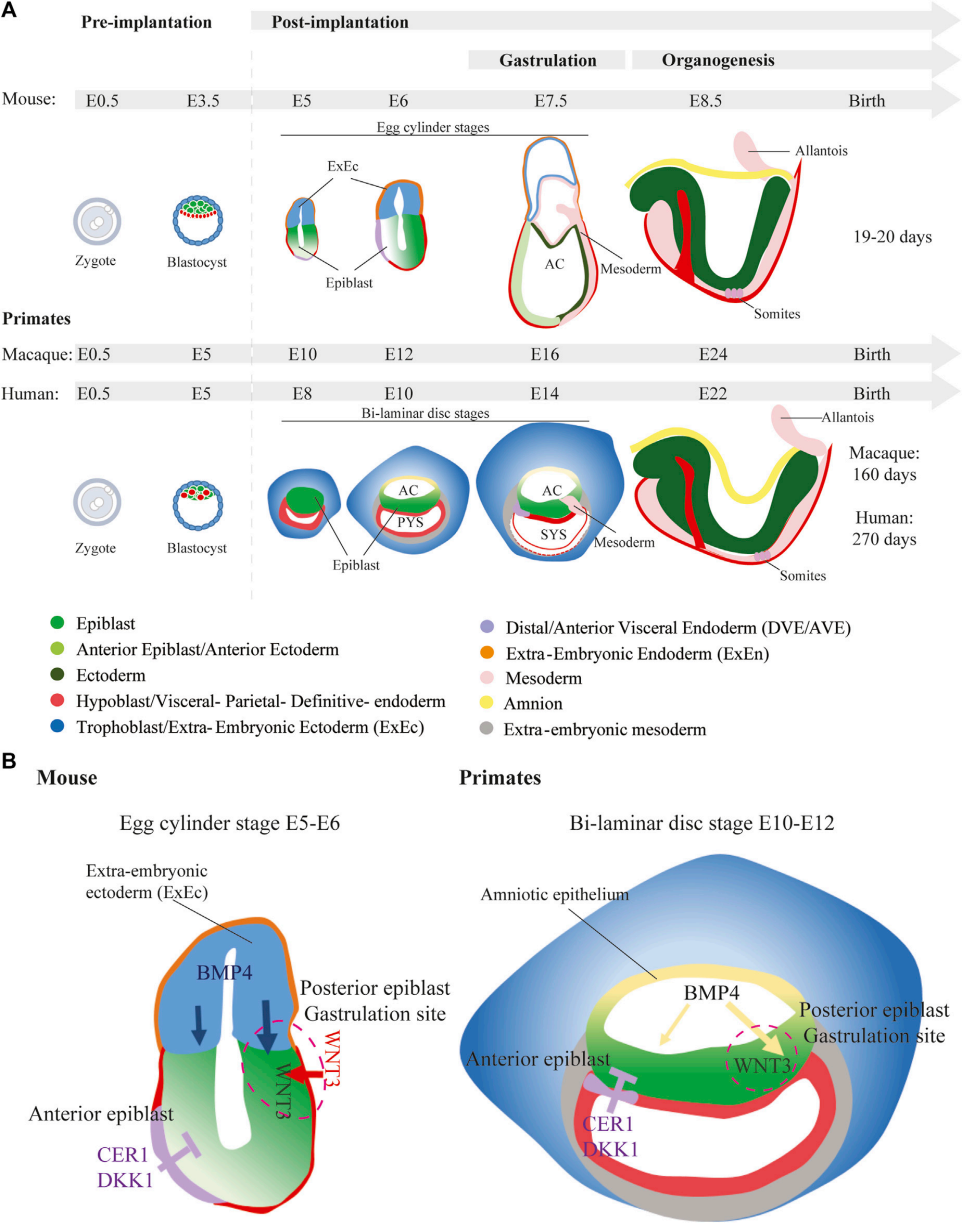
Embryonic development in mice *versus* primates. **(A)** Embryonic development of mice and primates. Mice go through egg cylinder stages of development, whereas primates form a bi-laminar disc. E, Embryonic days; AC, Amniotic Coelom; PYS, Primary Yolk Sac; SYC, Secondary Yolk Sac **(B)** Signaling mechanism inducing gastrulation in mice and in primates. In mice the source of BMP4 is the ExEc, whereas in primates the amnion produces BMP4.

Preimplantation development, spanning fertilization to implantation, is a crucial phase in early mammalian embryogenesis, marked by several key milestones. These key milestones include zygotic genome activation (ZGA), the transition to multicellularity through slow cell division, compaction, polarization, and subsequent blastocyst formation. Within the blastocyst, cells differentiate into the inner cell mass (ICM) and trophectoderm (TE), with further specification of the primitive endoderm (PrE; hypoblast) and epiblast within the ICM. Preimplantation development in mice spans 5 days, whereas in humans it generally takes 6–7 days. While mouse and human preimplantation development appears morphologically similar, differences emerge in cell fate specification, characterized by variations in the expression of lineage specific transcription factors and the activity of signaling pathways. After implantation, mouse and primate embryos clearly exhibit significant morphological and molecular differences. Lab mice have a gestation period of 19–20 days. In contrast, human gestation typically spans approximately 270 days, whereas in cynomolgus and rhesus monkeys this takes around 160 days each (Nakamura et al., 2021) (Figure 1A).

Following implantation, cell proliferation markedly increases in mouse embryos. This is accompanied by epithelialization of both the epiblast and the polar TE, leading to the formation of the characteristic cylindrical, elongated embryo, commonly referred to as the egg cylinder (Bedzhov et al., 2014a). The extra-embryonic ectoderm (ExEc), originating from the polar TE, plays a critical role in BMP4 production (Figure 1B). BMP4 is essential for establishing an anterior-posterior axis and initiating mesoderm formation in a NODAL dependent manner on the posterior side of the epiblast during gastrulation (Winnier et al., 1995; Lawson et al., 1999). After exposure to BMP4, WNT3 is first produced in the posterior visceral endoderm (VE) (Liu et al., 1999) before also appearing in the posterior epiblast (Rivera-Perez and Magnuson, 2005) (Figure 1B). Simultaneously, VE cells located at the anterior side of the egg cylinder are replaced by the anterior VE (AVE) cells which are initially formed at the distal tip of the egg cylinder (Thomas and Beddington, 1996; Thomas et al., 1998). The AVE, marked by transcription factors OTX2, HHEX, HESX1, FOXA2, and LHX1, acts as a protective barrier, preventing adjacent epiblast cells from responding to posteriorizing signals. The AVE does so by producing Wnt, Bmp, and Nodal antagonists like DKK1, CER1, and LEFTY1, thereby inhibiting ectopic primitive streak formation on the anterior side (Acampora et al., 1995; Belo et al., 1997; Thomas et al., 1998; Perea-Gomez et al., 1999; Kimura et al., 2000; Perea-Gomez et al., 2002; Yamamoto et al., 2004; Kimura-Yoshida et al., 2005) (Figure 1B).

In contrast, epiblast and TE cell proliferation in primate embryos, exhibits different morphological characteristics. The TE invades the endometrium, while the epiblast expands to form a flat sheet of cells, resulting in a flattened embryo known as an embryonic disc (Nakamura et al., 2016; Rossant and Tam, 2017; O'Rahilly and Muller, 2010). This early structure consists of two layers: the epiblast and the hypoblast. As development progresses, an amniotic cavity emerges following the separation of the amniotic epithelium from the epiblast layer, while the primary yolk sac is formed by the hypoblast layer. This primate-specific morphogenesis also presents already an extraembryonic mesoderm (ExEM) lineage in the pre-gastrulation embryo whereas in mice the ExEM (including the allantois, and mesoderm component of amnion and yolk sac) develops during gastrulation. However, the origin of the primate ExEM is unclear; it could have an epiblast origin as in mice, but could also be derived from the TE or the hypoblast (reviewed in (Pham et al., 2022; Rossant and Tam, 2022)). Also, in contrast to mice, where the ExEc serves as the signaling source for inducing gastrulation; in primates, BMP4 originates from the amnion (Figure 1B). Staining and profiling of *in vivo* non-human primate embryos indeed revealed the accumulation of BMP4 in the amnion of pre-gastrulating embryos (Sasaki et al., 2016; Bergmann et al., 2022). Similarly to mice, *WNT3*/WNT3 is detected in the non-human primate posterior epiblast (Niu et al., 2019; Bergmann et al., 2022). In cynomolgus monkeys, OTX2, along Wnt and Nodal inhibitors DKK1 and CER1, are detected in the AVE (Sasaki et al., 2016; Ma et al., 2019). In the human peri-implantation embryo, and similar to mice, the putative AVE exhibits an accumulation of CER1 and LEFTY1. At the gene expression level, the human AVE also presents an accumulation of *LHX1*, *HHEX* and *DKK1*-transcripts (Mole et al., 2021). *In vitro* experiments using human pluripotent stem cells (PSCs) have shown that the amnion is the source of BMP4 in primates and that BMP4 induces gastrulation in a WNT-dependent manner (Shao et al., 2017a; Zheng et al., 2019; Yang et al., 2021). It seems therefore that the formation of the primitive steak in mice and primates depends on the same signaling pathways.

These variations in development underscore substantial differences in early post-implantation developmental processes between mice and primates. Thus, making direct assumptions about human embryogenesis is challenging if based solely on the knowledge obtained from mouse development. Ethical restrictions will continue to limit the study of human embryogenesis. Using either non-human primate embryos or primate stem cell-derived embryo models holds the potential to provide valuable insights into the intricacies of human early embryonic development, effectively bridging the gap between mouse and human.

## Lineage emergence during preimplantation development

During the preimplantation phase, the TE, the epiblast, and the PrE are the first three lineages to emerge. The TE plays a role in forming the embryonic part of the placenta, while the epiblast gives rise to the embryo proper and the mesoderm of the allantois, amnion, and yolk sac. The PrE is vital for embryo patterning and contributes to the development of the yolk sac.

In mice, the major ZGA at the 2-cell stage (Schultz, 1993) marks the initiation of the expression of genes encoding various lineage specific transcription factors (TFs). The first lineage commitment in mice occurs with the specification of the TE from the outer cells of the morula stage embryo. This commitment is highly dependent on the expression of *Cdx2* initiated between the 8- and 16-cell stage (Niwa et al., 2005; Strumpf et al., 2005; Dietrich and Hiiragi, 2007; Ralston and Rossant, 2008; Jedrusik et al., 2010; Ralston et al., 2010) and on the inhibition of the Hippo signaling pathway (Yagi et al., 2007; Nishioka et al., 2008; Nishioka et al., 2009). Hippo-pathway inhibition in the TE cells results in the release of cytoplasmic sequestration of TEAD co-activator YAP1, enabling TE-specific genes like *Cdx2* and *Gata3* to be transcribed (Yagi et al., 2007; Nishioka et al., 2008). In comparison to mice, the major ZGA in humans occurs at the 4 to 8-cell stage (Braude et al., 1988; Blakeley et al., 2015). Human TE specification seems to be more dependent on *GATA3* than on *CDX2*. GATA3 can be detected in all cells of the morula stage human embryo, but subsequently becomes restricted to the TE (Petropoulos et al., 2016; Gerri et al., 2020), whereas CDX2 only appears well beyond the formation of the human blastocyst (Niakan and Eggan, 2013). Although the TE specifiers TEAD1, YAP and GATA3 predominantly co-localize in polarized outer cells of human 16-cell morulae, their presence is also detected in certain cells of compacting embryos before the establishment of cell polarity (Regin et al., 2023). This observation lends support to the idea that the initiation of TE formation in humans might occur independently of cell polarity. The observation that a genomic deletion of *TEAD4* does not affect the GATA3 profile in blastocyst-stage human embryos, unlike CDX2, supports that notion (Stamatiadis et al., 2022). Cell polarity therefore influences the TE lineage formation in humans, but its importance in TE specification appears to be less evident than in mice (Gerri et al., 2020; Zhu et al., 2021).

The second cell fate decision in preimplantation embryos involves the specification of the PrE and the epiblast in ICM cells. The murine ICM is composed of a random mixture of cells in a “salt and pepper” pattern displaying varying levels of GATA6 or NANOG (Chazaud et al., 2006). Ultimately FGF produced by epiblast-biased cells expressing *Nanog,* support further specification of murine PrE-biased cells (Silva et al., 2009; Messerschmidt and Kemler, 2010; Yamanaka et al., 2010; Frankenberg et al., 2011). Without sufficient FGF signaling, PrE-biased cells revert back to the epiblast fate. This mechanism most likely ensures a perfect balance between the number of PrE and epiblast cells. This same second cell fate decision in human embryos is less well understood, and differs from its murine counterpart as FGF signaling does not seem to be as essential for human PrE development (Kuijk et al., 2012; Roode et al., 2012). Additionally, detection of TFs POU5F1, SOX2 and NANOG generally associated with the pluripotent epiblast, also differs between mouse and human preimplantation embryos. In mouse, SOX2 is the first pluripotency TF to selectively mark the epiblast (Frum and Ralston, 2015), whereas in human this role is reserved to NANOG (Cauffman et al., 2009). Variations can also be observed in the KLF family members expression profiles. As such, while *Klf2* expression delineates the mouse epiblast, *KLF17* expression performs a similar role in the human epiblast (Blakeley et al., 2015; Lea et al., 2021).

## Cellular plasticity in preimplantation development

In the intricate nomenclature hierarchy of cellular potential, totipotent cells stand at the top. Totipotency can be interpreted as the capacity of a single diploid cell to independently give rise to an entire organism. Alternatively, a more lenient interpretation considers totipotency as the capacity of a cell to differentiate into all types of lineages, in amniotes including all embryonic and extra-embryonic lineages. To delineate these distinctions, the term “totipotency” has been suggested for the former, emphasizing organism-forming ability, while the term “plenipotency” has been proposed for the latter (Condic, 2014). Pluripotent cells, on the other hand, while highly versatile, are confined to producing derivatives specific to the epiblast (for a discussion of used functional assays for pluripotency and their limitations, with mouse and human cells, see (De Los Angeles et al., 2015)). Despite their adaptability, they lack the organizational finesse required to forming an integrated body plan and can generate neither TE nor PrE derivatives. In the strict sense of the word, a fertilized oocyte is classified as totipotent. However, due to ethical considerations, the developmental potential of individual human blastomeres to give rise to viable offspring has never been assessed. In mice, blastomeres of 2-cell stage embryos can be categorized as totipotent because a single blastomere from the 2-cell stage embryo, albeit with a lower potency, is sufficiently competent to produce offspring (Tarkowski, 1959; Tsunoda and McLaren, 1983; Lawitts and Graves, 1988; Papaioannou et al., 1989; Papaioannou and Ebert, 1995; Wang et al., 1997; Sotomaru et al., 1998; Morris et al., 2012b; Casser et al., 2017; Rahbaran et al., 2022). Four-cell stage embryos are generally assumed not be totipotent anymore (Tarkowski and Wroblewska, 1967; Rossant, 1976; Morris et al., 2012b) although at least one report describes offspring from a single blastomere removed from 4-cell stage embryos (Maemura et al., 2021). Whereas a critical aspect of this potency loss is due to an insufficient number of cells forming the epiblast (Rossant, 1976; Morris et al., 2012b), resulting in smaller sized offspring (Tsunoda and McLaren, 1983; Morris et al., 2012b; Casser et al., 2017), blastomeres from 2- and 4-cell stage mouse embryos have also been shown to possess differing developmental potentials (Piotrowska-Nitsche et al., 2005; Torres-Padilla et al., 2007; Morris et al., 2012b). This implies that lineage specification, involving the initial commitment of cells to specific fates starts already at the 2-cell stage. Nevertheless, blastomeres from mouse cleavage stage embryos retain the plasticity to contribute to all lineages and are not yet committed to one specific fate. Even cells within the ICM of early blastocyst-stage mouse embryos (E3) retain plenipotency, capable of producing both the trophectoderm (TE) and the primitive endoderm (PrE) in addition to the epiblast (Handyside, 1978; Hogan and Tilly, 1978; Spindle, 1978; Rossant and Lis, 1979; Suwinska et al., 2008; Wigger et al., 2017). However, TE cells lose the competency to produce the ICM once a blastocyst is formed (Suwinska et al., 2008). In mice, the ICM loses its potency to form the TE after the second cell fate decision, with the specification of the PrE and the pluripotent epiblast (Posfai et al., 2017; Wigger et al., 2017). Blocking this second cell fate decision using small molecules specifically targeting FGF/ERK signaling, preserves the potency of the murine ICM to form TE (Wigger et al., 2017). Plenipotency during human embryonic development is maintained longer than observed in mice. Both the TE and ICM of the human early blastocyst-stage embryo retain the potency to form all lineages, as demonstrated in an embryo dissociation study (De Paepe et al., 2013). Additionally, the human naïve epiblast maintains its potential to form TE and PrE (Guo et al., 2021). Pseudotime analysis of single cell transcriptomics in mouse embryos confirms two sequential lineage determination events, with TE-specific cells emerging at the morula stage, and PrE-specific cells emerging in the ICM of the blastocyst stage embryo (Meistermann et al., 2021). A similar analysis in human embryos also identifies two lineage determination events, but both occur sequentially after the blastocyst is formed (Meistermann et al., 2021), albeit that an earlier study suggested that the first and second lineage segregation occur simultaneously (Petropoulos et al., 2016). It seems, thus, that the morphological appearance of the blastocyst in humans does not fully align with the molecular divergence.

## Murine early lineage stem cells

**FIGURE 2:**
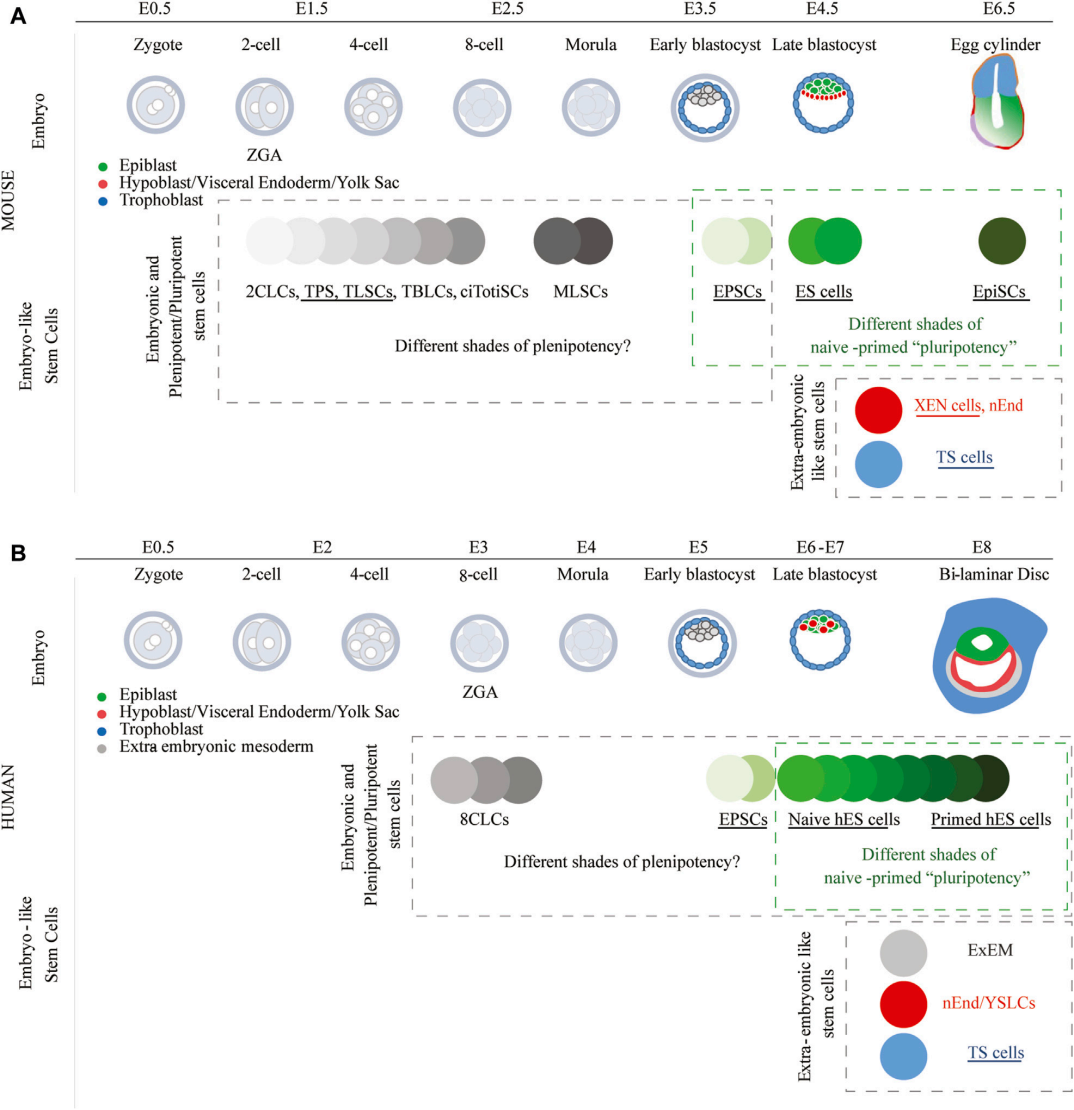
Stem cells resembling early embryonic lineages in mice and humans. Many of these stem cell types have been derived from existing ES cells by modifying culture conditions. Following the implementation of these cell culture conditions, certain studies have also shown that specific cell types could be directly derived from the embryo (underlined stem cell types). **(A)** Since 2CLCs, TPS cells, TLSCs, TBLCs, ciTotiSCs, MLSCs, and EPSCs can form both embryonic and extra-embryonic murine lineages, these cell types can be classified as plenipotent. On the other hand, EPSCs could also be classified as pluripotent as they have limited potential to form the mouse trophoblast. **(B)** Whereas naïve and primed human ES cells are classified as pluripotent, they could also, together with 8CLCs and EPSCs, be classified as plenipotent, as all can form the embryonic and extra-embryonic cell lineages.

The first murine embryonic stem (ES) cells were isolated in 1981 (Evans and Kaufman, 1981; Martin, 1981). These cells are derived from the epiblast of blastocyst-stage embryos and have the capacity to contribute to all embryonic lineages when injected into murine preimplantation embryos, but these cells rarely contribute to extra-embryonic tissues (Bradley et al., 1984; Beddington and Robertson, 1989). The unrestricted potential to produce all embryonic lineages is the defining trait of pluripotency, a trait which is maintained after implantation of the blastocyst in the uterus up until the epiblast starts gastrulation. Whereas, initially PSCs were derived from the epiblast of preimplantation embryos (Evans and Kaufman, 1981; Martin, 1981), they could also be derived from the post-implantation epiblast (Brons et al., 2007; Tesar et al., 2007). The epiblast-derived PSCs of the pre- and post-implantation embryo were labeled as naïve and primed PSCs (Figure 2A), respectively (Nichols and Smith, 2009). Primed PSCs are also frequently named epiblast stem cells (EpiSC). Naïve ES cells grow as small, compact, domed colonies; while primed ES cells grow as flat epithelialized colonies. Whereas the primed PSCs can contribute to the epiblast when injected into a post-implantation embryo, they are developmentally too advanced to efficiently contribute to offspring when injected into recipient preimplantation embryos (Brons et al., 2007; Tesar et al., 2007; Huang et al., 2012). This makes the generation of chimeric mice using primed PSCs technically challenging.

Furthermore, also mouse trophoblast stem (TS) cells (Tanaka et al., 1998; Kubaczka et al., 2014; Ohinata and Tsukiyama, 2014) and mouse eXtra-embryonic ENdoderm (XEN) stem cells (Kunath et al., 2005; Zhong et al., 2018) have subsequently been derived from the mouse TE and PrE, respectively (Figure 2A). Following injection into preimplantation embryos, TS cells and XEN cells contribute exclusively and respectively to the trophoblast and extra-embryonic endoderm lineages. Whereas ES cells can in adapted culture conditions transdifferentiate to trophoblast (Hayashi et al., 2010) as well as extra-embryonic endoderm (Cho et al., 2012; Niakan et al., 2013; Anderson et al., 2017), the overexpression of lineage-specific transcription factor (TF) (trans)genes greatly facilitates this conversion. As such, induced expression of trophoblast TF (trans)genes (e.g., *Cdx2* and *Gata3*) supports a transformation towards TS cells (Niwa et al., 2000; Niwa et al., 2005; Kuckenberg et al., 2010; Ralston et al., 2010; Cambuli et al., 2014; Wei et al., 2016; Kaiser et al., 2020); whereas the induced expression of PrE TF (trans)genes (e.g., *Gata6* and *Sox17*) supports the formation of mouse XEN cells (Fujikura et al., 2002; Shimoda et al., 2007; Shimosato et al., 2007; Qu et al., 2008; Niakan et al., 2010; McDonald et al., 2014; Wamaitha et al., 2015; Wei et al., 2016). Nevertheless, epigenetic barriers, such as methylation of the *Elf5* promoter in ES cells, hamper generally a complete transdifferentiation of ES cells into TS cells (Ng et al., 2008; Hemberger, 2010; Cambuli et al., 2014).

Scientists have therefore been captivated by creating a plenipotent stem cell that could contribute to the embryonic and extra-embryonic tissues. Three types of plenipotent stem cells have been described, i.e., 2-cell like stem cells (2CLCs), expanded/extended potential stem cells (EPSCs) and morula like stem cells (MLSCs). The 2CLCs were discovered as a small (less than 1%) transient population in ES cell cultures (Macfarlan et al., 2012). The 2CLCs present characteristics of blastomeres of the 2-cell stage embryo when major ZGA occurs, and their population size can be increased by modulating culture media and/or overexpression of specific (trans)genes (Macfarlan et al., 2011; Hisada et al., 2012; Macfarlan et al., 2012; Maksakova et al., 2013; Dan et al., 2014; Lu et al., 2014; Hayashi et al., 2016; Choi et al., 2017; Rodriguez-Terrones et al., 2018; De Iaco et al., 2019; Yan et al., 2019; Hu et al., 2020; Rodriguez-Terrones et al., 2020; Huang et al., 2021; Olbrich et al., 2021; Shen et al., 2021; Wang et al., 2021; Xu et al., 2022; Yang et al., 2022; Hu et al., 2023; Meharwade et al., 2023). Cells presenting more or less characteristics of the 2-cell stage embryo have been labeled in the literature as 2-cell-like cells (2CLCs), but also as totipotent blastomere-like cells (TBLCs), totipotent-like stem (TPS) cells, totipotent-like stem cells (TLSCs) and chemically induced totipotent stem cells (ciTotiSCs). These cell types seem to be more potent than ES cells as they can contribute to all extra-embryonic lineages when injected into preimplantation embryos (Macfarlan et al., 2012; Choi et al., 2017; Shen et al., 2021; Wang et al., 2021; Xu et al., 2022; Yang et al., 2022; Meharwade et al., 2023). In this manuscript, the cell types presenting some characteristics of the 2-cell stage are all compiled under 2CLC terminology (Figure 2A). 2CLCs should however not be confounded with EPSCs. Murine EPSCs which can be obtained by modifying culture media composition, have also the potential to form all extra-embryonic lineages (Yang et al., 2017a; Yang et al., 2017b; Yang et al., 2019). A thorough assessment of EPSCs, however, labels EPSCs to be more similar to a blastocyst than a pre-blastocyst stage embryo and their capacity to differentiate into trophoblast has been questioned (Stirparo et al., 2018; Posfai et al., 2021). MLSCs, which would relate to the morula-stage embryo, have recently also been described (Li et al., 2023a). Whereas the authors reported that MLSCs are plenipotent and can form the extra-embryonic lineages, the formation of the trophoblast seemed difficult as observed during the formation of blastoids.

An analytical exploration that highlights the commonalities among embryos at different developmental stages and certain *in vitro* cell types recognized for their enhanced developmental potential (2CLCs, TBLCs, TPS, and EPSCs) reveals that the analyzed cell types all display a predominant association with morula and pre- and post-implantation epiblast stages (Xu et al., 2022). Are all these alleged murine ‘plenipotent’ cell types fundamentally dissimilar, or are they merely a product of *in vitro* adaptation to distinct culture conditions or the result of overexpressed (trans)genes? A larger integration of diverse datasets obtained from single-cell RNA sequencing across various stages of preimplantation embryos, including ES cells, EpiSCs, various cell types clustered under 2CLC type in this manuscript, as well as EPSCs and MLSCs, would be extremely welcome to correlate them better with a specific developmental stage (Figure 2A).

## Human early lineage stem cells

Human ES (hES) cells were derived over a decade ago, following the establishment of germline-competent mouse ES cell lines (Thomson et al., 1998). Unlike mouse ES cells, which were isolated and maintained in cell culture in their naive state, conventional hES cells resemble the pre-gastrulation epiblast and are thus in the primed pluripotent state (Nakamura et al., 2016). It has been suggested that the naïve ES cells from mice rely on signaling pathways governing murine diapause (Nichols et al., 2001), a reversible state of suspended embryonic development that allows blastocyst-stage embryos to delay implantation until favorable environmental conditions arise. In contrast, human development is continuous, and lacks a natural mechanism like diapause to halt blastocyst development without compromising viability.

The delayed onset of ZGA in humans at the 8-cell stage (Braude et al., 1988; Blakeley et al., 2015), as opposed to the 2-cell stage in mice (Schultz, 1993); is expected to influence lineage specification and modify the signaling pathways governing this process (Rossant and Tam, 2017). Although several culture media have been described to support the culture of naïve human PSCs (Gafni et al., 2013; Takashima et al., 2014; Theunissen et al., 2014; Duggal et al., 2015; Qin et al., 2016; Zimmerlin et al., 2016; Guo et al., 2017; Bredenkamp et al., 2019; Bayerl et al., 2021; Yu et al., 2022; Ai et al., 2023), the precise nature of true naïve pluripotency remains elusive. All human naïve pluripotency cell culture media likely represent different shades of pluripotency between true naïve and primed pluripotency (Figure 2B). To date, 5iLAF (Theunissen et al., 2014), t2iLGö (Takashima et al., 2014; Guo et al., 2017) and PXGL (Bredenkamp et al., 2019) are considered the naive and complex culture systems that produce naive hES cells representing the pre-implantation epiblast (Stirparo et al., 2018). Naive hES cell cultures exhibit considerable heterogeneity, presenting 8-cell-like cells (8CLCs) with features of ZGA (Wang et al., 2018; Mazid et al., 2022; Moya-Jódar et al., 2023; Taubenschmid-Stowers et al., 2022; Yoshihara et al., 2022; Yu et al., 2022) as well as TE- and PrE-like cells (Linneberg-Agerholm et al., 2019; Dong et al., 2020; Moya-Jódar et al., 2023). The human preimplantation epiblast retains the plasticity to form trophoblast and PrE after the blastocyst has been formed (De Paepe et al., 2013; Guo et al., 2021) which is likely associated with the naïve state of the epiblast. Since the human preimplantation epiblast maintains naïve characteristics at least until E7 (Messmer et al., 2019), it could be assumed that this plasticity extends at least until that day. Reflecting this plasticity, naïve hES cells besides having the capacity to form EPSCs (Yang et al., 2017b; Gao et al., 2019; Liu et al., 2021a) can also be easily directed towards the extra-embryonic lineages including TS cells (Xu et al., 2002; Cinkornpumin et al., 2020; Dong et al., 2020; Io et al., 2021; Mischler et al., 2021; Wei et al., 2021; Viukov et al., 2022), hypoblast-like stem cells (Linneberg-Agerholm et al., 2019; Mackinlay et al., 2021) and ExEM (Pham et al., 2022) (Figure 2B).

Human extra-embryonic stem cell lines, however, are not as well characterized as their murine counterparts. Human TS cells can be directly derived from embryos (Okae et al., 2018) or transdifferentiated from hES cells (Xu et al., 2002; Cinkornpumin et al., 2020; Dong et al., 2020; Io et al., 2021; Mischler et al., 2021; Wei et al., 2021; Viukov et al., 2022). Human hypoblast-like stem cells, named naïve endoderm (nEnd) and yolk like stem cells (YLSCs), on the other hand, have been exclusively obtained following differentiation of hES cells (Linneberg-Agerholm et al., 2019; Mackinlay et al., 2021). Several studies have suggested that differentiation towards the early extra-embryonic lineages (TE and PrE) is only possible when using naïve hES cells, but impossible when using primed hES cells (Linneberg-Agerholm et al., 2019; Dong et al., 2020; Guo et al., 2021; Io et al., 2021). However, other studies claim that early extra-embryonic lineages can be formed from primed hES cells (Xu et al., 2002; Wei et al., 2021; Viukov et al., 2022). Primed hES cells, representing the post-implantation epiblast, are very responsive to somatic differentiation cues (e.g., BMP4, ACTIVIN) unlike naïve hES cells that do not respond well to somatic differentiation cues and prefer to be re-primed before differentiation (Guo et al., 2017; Liu et al., 2017; Rostovskaya et al., 2019). It is worth considering whether primed hES cells retain the capacity to generate early lineages while also displaying responsiveness in forming somatic cell derivatives, which could potentially result in the overgrowth of the initially formed early lineage cell types. Unlike murine extra-embryonic stem cells, human extra-embryonic stem cells require intricate culture media for maintenance. The true nature of human extra-embryonic stem cell lines is still unclear. Although human TS cells serve as valuable models for investigating trophoblast differentiation, they are generally regarded as having closer associations with post-implantation cytotrophoblasts than with the TE of the blastocyst (Okae et al., 2018; Castel et al., 2020; Mischler et al., 2021). Thus far, no PrE stem cell line has been directly established from human embryos. A better characterization of how the PrE emerges in the human embryo may provide valuable insights on how to generate a hypoblast-like stem cell line directly derived from embryos. Nevertheless, it seems that naïve hES cells are sufficiently plenipotent to form the early lineages emerging during development. Culture procedures for growing naïve hES cells, however, still require further refinement.

## The blastoid or pre-implantation integrated stem cell-based embryo model

**FIGURE 3:**
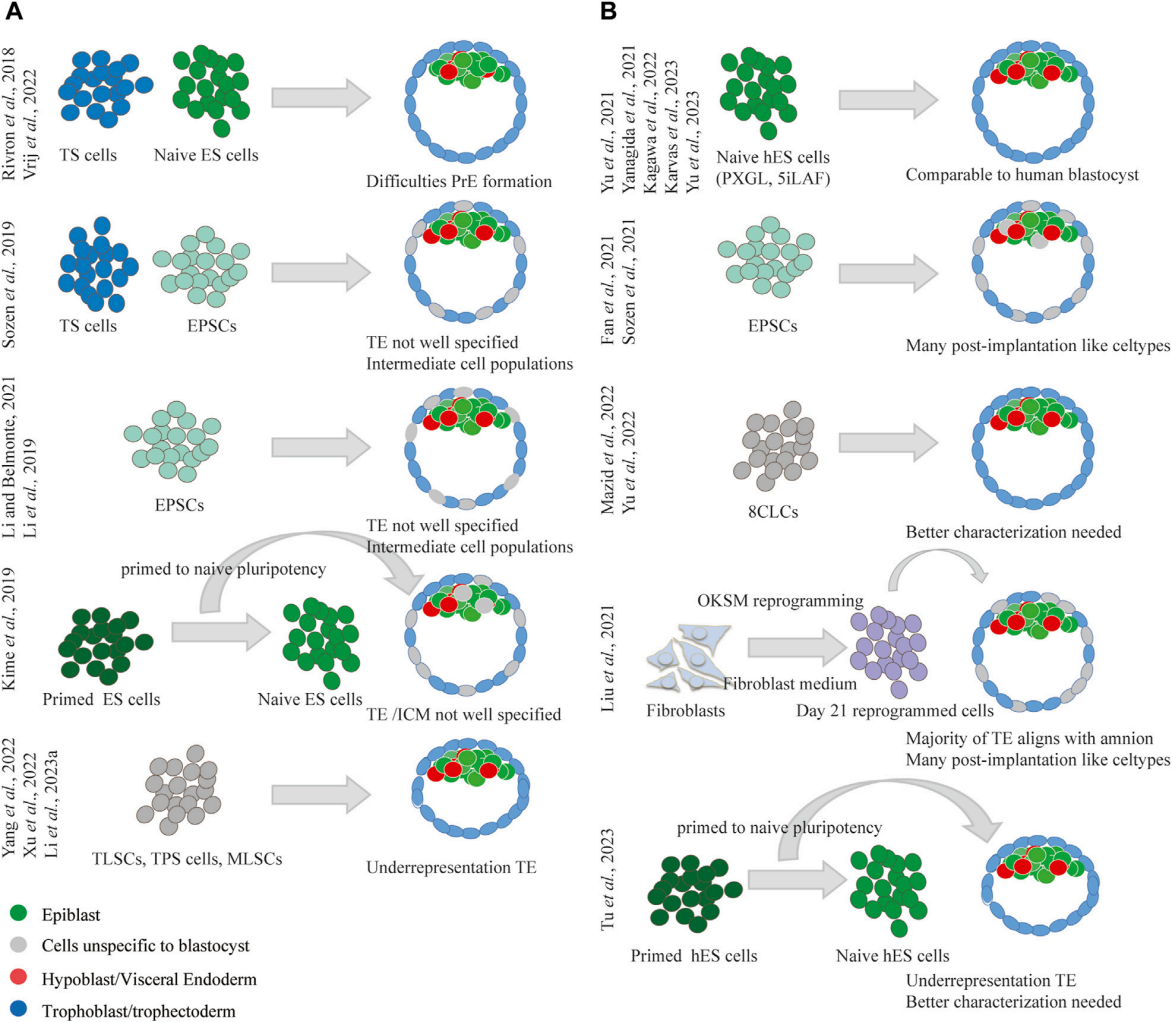
Summary of strategies used for blastoid formation in mouse and human. **(A)** Diverse strategies have been employed to produce mouse blastoids, all struggle with the formation of the extra-embryonic lineages. **(B)** Diverse strategies have been employed to produce human blastoids, naïve human ES cells as starting cell type showed the best capacity to produce blastocyst alike blastoids.

The blastoid, which mimics the blastocyst stage, has been successfully generated in mouse (Yang et al., 2017b; Rivron et al., 2018; Li et al., 2019; Sozen et al., 2019; Li and Izpisua Belmonte, 2021; Vrij et al., 2022; Xu et al., 2022; Yang et al., 2022; Li et al., 2023a) by assembling one or two types of stem cells *in vitro* (Figure 3A). While there have been reports of mouse iBlastoids forming spontaneously during the reprogramming of primed to naïve states (Kime et al., 2019), this strategy does not allow robust and high-throughput blastoid production, and their trophectoderm is not well defined. The initial study detailing the formation of murine blastoids used ES cells in combination with TS cells (Rivron et al., 2018). A notable challenge encountered was the underrepresentation of the PrE in these structures as the used ES cells did not naturally form PrE-like cells. To address this issue, the protocol was chemically modified to stimulate ES cell lines to differentiate into PrE, contributing to the formation of blastoids better featuring PrE (Vrij et al., 2022). In alternative approaches, researchers have sought to leverage the increased developmental potential of EPSCs, 2CLCs and MLSCs, to generate blastoids (Yang et al., 2017b; Li et al., 2019; Sozen et al., 2019; Li and Izpisua Belmonte, 2021; Xu et al., 2022; Yang et al., 2022; Li et al., 2023a). Protocols were devised to exclusively use EPSCs (Li et al., 2019; Li and Izpisua Belmonte, 2021) or combine EPSCs with TS cells (Sozen et al., 2019). While blastoids formed using EPSCs morphologically resembled blastocysts and exhibited the correct distribution of lineage markers across all three early lineages of the blastocyst, including the PrE. Detailed single-cell transcriptomic analyses revealed inadequate specification of the TE alongside the presence of undefined intermediate, mesoderm-like populations when EPSCs were part of the aggregation protocol (Posfai et al., 2021). Three additional studies reporting on the formation of stem cells with plenipotent traits, namely, TLSCs (2CLC-like) (Yang et al., 2022), TPS cells (2CLC-like) (Xu et al., 2022) and MLSCs (Li et al., 2023a), demonstrated the competency of these cells to form blastoids. While these blastoids appeared to exhibit the early three lineages (TE, PrE, and epiblast) similar to blastocysts, a comprehensive analysis was not the main focus of these studies. However, an under-representation of TE cells was observed in blastoids generated from TPS cells (Xu et al., 2022) and MLSCs (Li et al., 2023a).

Mouse blastoids thus present difficulties in forming extra-embryonic lineages. While ES cells face challenges with PrE formation; EPSCs, 2CLCs, and MLSCs struggle with TE specification. This also partially elucidates the limited capacity of mouse blastoids to develop further *in vitro* and *in vivo*. As authentic blastocysts also encounter hurdles in transitioning to post-implantation development *in vitro*, there is a pressing need for the enhancement of culture platforms supporting the post-implantation development of blastocysts to more accurately assess the developmental potential of blastoids.

Various approaches have been used in the development of human blastoids, employing different cell types as starting materials (Figure 3B). Human blastoids have been successfully generated using hES cells in both the naive (Yanagida et al., 2021; Yu et al., 2021; Kagawa et al., 2022; Karvas et al., 2023; Yu et al., 2023) and primed-to-naive intermediate pluripotency states (Tu et al., 2023), as well as EPSCs (Fan et al., 2021; Sozen et al., 2021) and 8CLCs (Mazid et al., 2022; Yu et al., 2022). Moreover, iBlastoids have been observed to form during the reprogramming of fibroblasts into a pluripotent state (Liu et al., 2021b). Thus far, there have been no reports of human blastoid formation using human TS cells (Okae et al., 2018). This may be explained by the closer similarity of human TS cells to post-implantation cytotrophoblasts rather than TE (Okae et al., 2018; Castel et al., 2020; Mischler et al., 2021). While certain studies have outlined some *in vitro* post-implantation development following 2D culture, human blastoids typically do not easily transition into this stage. This limitation may be partially attributed to the presence of cell types more related to post-implantation development (ExEM, amnion-like, definitive endoderm, etc. (Pham et al., 2022; Zhao et al., 2024)), particularly evident in iBlastoids (Liu et al., 2021b) and blastoids formed from EPSCs (Fan et al., 2021; Sozen et al., 2021). In general, blastoids derived from naive hES cells appear to align better with the blastocyst (Yanagida et al., 2021; Yu et al., 2021; Kagawa et al., 2022; Karvas et al., 2023; Yu et al., 2023) and present fewer cell types characteristic of advanced developmental stages (Zhao et al., 2024). Blastoids formed from naive hES cells thus have the best capacity to display *in vitro* post-implantation development, with 3D matrices further augmenting this potential (Karvas et al., 2023; Tu et al., 2023). Blastoids generated from 8CLCs have also been described, but their thorough analysis to assess lineage alignment with human embryonic pre- or post-implantation stages is pending.

Unlike in mice, where the formation of extra-embryonic lineages poses challenges, human blastoids demonstrate the ability to form these lineages using solely hES cells or their derivatives such as EPSCs (Fan et al., 2021; Sozen et al., 2021) and 8CLCs (Mazid et al., 2022; Yu et al., 2022). This distinction is attributed to the plenipotent plasticity of the human stem cells, which facilitates the human blastoid protocol but also introduces unintended or undesired cell types. Further research efforts and refinements of human blastoid generation protocols are essential to overcome current limitations and enhance their accuracy and efficiency as models for both pre- and post-implantation development. While optimizing post-implantation culture protocols can enhance the *in vitro* development of blastoids, assessing *in vivo* development of human blastoids will remain ethically restricted (Hyun et al., 2020). To evaluate the implantation potential of blastoids, both 2D and 3D endometrial cell cultures can be used to model the attachment and invasion of blastoids into the endometrium. For instance, blastoids can be seeded onto an open-faced endometrial layer obtained from endometrium organoids cultured in 2D to simulate attachment to the endometrium (Kagawa et al., 2022). Alternatively, recent advancements in 3D endometrial models offer promising alternatives to study not only adhesion but also invasion of blastocysts into the endometrium (Shibata et al., 2024). Furthermore, the recent report of non-human primate blastoid generation (Li et al., 2023b), offers an avenue to evaluate the *in vivo* implantation and developmental potential of blastoids in non-human primate species.

## Optimizing culture conditions for extended embryo culture using real embryos

To develop effective models for studying post-implantation embryogenesis in both mice and humans, prioritizing the optimization of culture conditions first with authentic embryos is essential. However, creating an environment that faithfully mirrors the intricacies of human embryonic development proves more challenging than in mice. In mice, implantation occurs through the mural TE, co-explaining the typical egg cylinder shape. In humans, trophoblast invasion is initiated by the polar TE, leading to the formation of a bi-laminar disc shape (Weberling and Zernicka-Goetz, 2021). In mice, the polar TE independently develops into an extra-embryonic ectoderm, playing an instructive role in epiblast development without direct dependence on the endometrium. Conversely, human epiblast development relies more on interaction with the endometrium. Adding to the complexity is the inherently slower pace of human embryonic development, likely increasing the challenges associated with culture procedures. While *in vitro* conditions are advancing, they can never replicate the finely tuned environment of the uterine womb perfectly. In fact, the longer the span of *in vitro* culture, the greater the risk of inducing aberrations in embryonic development (Sunde et al., 2016).

Decades ago, numerous studies documented the *in vitro* growth of mouse preimplantation embryos, extending up to somite and limb bud stages (Hsu, 1971; Hsu, 1973; Hsu et al., 1974; Pienkowski et al., 1974; Chen and Hsu, 1982; Tachi, 1992). Remarkably, this research was revived nearly 40 years later, initially describing the development of preimplantation embryos up to the egg cylinder stages (Morris et al., 2012a; Bedzhov et al., 2014b; Bedzhov and Zernicka-Goetz, 2014). Subsequent modifications refined the culture procedures, enabling development again up to embryonic day 11 (E11) (Aguilera-Castrejon et al., 2021; Aguilera-Castrejon and Hanna, 2021). However, significant opportunities for further optimization exist. Mouse embryos retrieved at the pre-implantation stage (E3.5-E4.5) rarely progress through *in vitro* development, and only up to 20% of embryos retrieved at the early post-implantation stage (E5.5) can develop until a stage comparable to E11.5 (Aguilera-Castrejon and Hanna, 2021). The challenge in supporting the development of preimplantation embryos to such advanced stages arises at least partially from the need for these stages to undergo a cell adhesion phase on a 2D surface, disrupting normal embryonic morphogenesis. Consequently, various research groups have invested in developing 3D platforms that would mimic the uterine environment. Recently published studies suggest promising outcomes in these 3D platforms (Gu et al., 2022; Ichikawa et al., 2022; Bondarenko et al., 2023). In one study, it was demonstrated that 11% of retrieved mouse preimplantation embryos developed up to early organogenesis when cultured in a 3D platform (Gu et al., 2022). At E11.5, the placenta is necessary to distribute nutrients to the fetal body and eliminate waste (Zhai et al., 2022). Thus, to extend such *in vitro* cultures, efforts will have to be undertaken to provide better support of placenta development.

Concurrently, culture conditions conducive to prolonged human embryo development have been established, allowing up to 31% of preimplantation embryos to progress until a stage resembling E14 (Lindenberg et al., 1985; Deglincerti et al., 2016; Shahbazi et al., 2016; Zhou et al., 2019; Xiang et al., 2020). Nevertheless, ethical constraints present considerable challenges to advancing human embryo culture, limiting our understanding of embryonic development beyond gastrulation and impeding the exploration of organogenesis in the human embryo (Warnock, 1985). Given these ethical challenges, the exploration of culture platforms for non-human primate embryos becomes a valuable alternative (Lopata et al., 1995; Ma et al., 2019; Niu et al., 2019; Gong et al., 2023; Zhai et al., 2023). Unlike human embryos, non-human primates offer a more permissive experimental environment, facilitating a more comprehensive exploration of embryonic development, particularly during stages beyond gastrulation and into organogenesis. Using similar culture conditions as those used for extended culture of human preimplantation embryos, 21%–35% of cynomolgus monkey embryos could be cultured until a stage resembling E19-E20 before collapsing, due to technical limitations (Ma et al., 2019; Niu et al., 2019). Remarkably, two recently introduced adapted 3D culture platforms have demonstrated the ability to sustain *ex utero* growth of 20%–34% of cynomolgus monkey embryos up to E25-like stage, thereby extending *ex utero* development until early organogenesis (Gong et al., 2023; Zhai et al., 2023). Similar to mice, efforts will have to be directed towards enhancing placental development to support prolonged *ex utero* development in primates.

## Partially and fully integrated stem cell-based embryo models mimicking early mouse post-implantation development

In contrast to blastoids, which rely on complex culture media for formation, the development of integrated post-implantation embryoids primarily depends on the interactive dynamics of employed stem cell lines or formed lineages to guide each other’s development.

In mouse embryonic development, the ExEc serves as the pivotal signaling center orchestrating germ layer formation. Experimental models, relying on the assembly of TS and ES cells, enable the simulation of early mouse post-implantation development. Initial studies formed self-organized bi-compartmental structures resembling the epiblast and ExEc observed in the egg cylinder, by simple aggregation of ES and TS cell suspensions (Harrison et al., 2017). Those egg cylinder-like aggregates (ETS models) exhibited anterior-posterior embryo polarity and demonstrated the emergence of mesoderm as well as Primordial Germ Cells (PGCs). A similar study, assembling pre-aggregated TS and ES cell aggregates, resulted in the formation of EpiTS embryoids. The authors noted that an egg cylinder displaying anterior-posterior polarity could only be obtained when the ES cell compartment displayed an epithelial-like morphology before the ES and TS cell aggregates were assembled (Girgin et al., 2021). Notably, both studies used Matrigel to induce epithelialization of the epiblast (Bedzhov and Zernicka-Goetz, 2014). *In vivo*, the basement membrane between the VE and the epiblast plays a crucial role in inducing epiblast cell polarization and lumenogenesis. The murine Embryonic-Trophoblast-eXtra embryonic endoderm (ETX) model circumvents the need for Matrigel by employing real extra-embryonic endoderm cells during aggregation. Mouse ETX models rely solely on the assembly of these three types of stem cells, with minimal interference from culture media. However, achieving optimal results requires precise titration of cell numbers to simulate normal, dose-dependent effects of signaling factors orchestrating embryogenesis and accurately model murine embryonic development. The initial model employing these three lineages for assembly used XEN cells as suppliers for the extra-embryonic endoderm (Sozen et al., 2018). However, this model did not progress beyond early gastrulation. A crucial enhancement for further development involved substituting XEN cells with ES cells expressing PrE-specific TF (trans)genes in an inducible manner (Amadei et al., 2021; Amadei et al., 2022; Lau et al., 2022; Tarazi et al., 2022; Dupont et al., 2023). The limited competence of XEN cells, resembling parietal endoderm (Kunath et al., 2005), likely explains their less favorable performance. Conversely, substituting TS cells with ES cells capable of forming TS cells through induced expression of trophoblast-specific TFs was not beneficial when compared to using authentic TS cells (Lau et al., 2022; Tarazi et al., 2022). Epigenetic barriers between TS and ES cell lineages (Ng et al., 2008; Hemberger, 2010; Cambuli et al., 2014) likely hinder ES cells from easily forming fully developed TS cells. Chimeric experiments have provided ample support regarding the developmental restrictions of ES, TS, and XEN cells (Bradley et al., 1984; Beddington and Robertson, 1989; Tanaka et al., 1998; Kunath et al., 2005; Kubaczka et al., 2014; Ohinata and Tsukiyama, 2014). During the assembly of ETX embryoids, the used stem cells also remain developmentally restricted, contributing exclusively to their lineage of origin (Dupont et al., 2023). This observation is crucial as it enables scientists to explore early lineage interactions using stem cell lines harboring targeted mutations in key developmental genes, thus addressing biological inquiries previously unattainable in *in vivo* embryos. Currently, the murine post-implantation model recapitulates embryonic development until a stage that strongly resembles E9 (Lau et al., 2022; Tarazi et al., 2022). Given the nuanced nature of developmental outcomes, optimizing the efficiency of ETX embryoid production (Dupont et al., 2023) is still essential to ensure an adequate supply of data points.

## Partially and fully integrated stem cell-based embryo models mimicking early human post-implantation development

**FIGURE 4:**
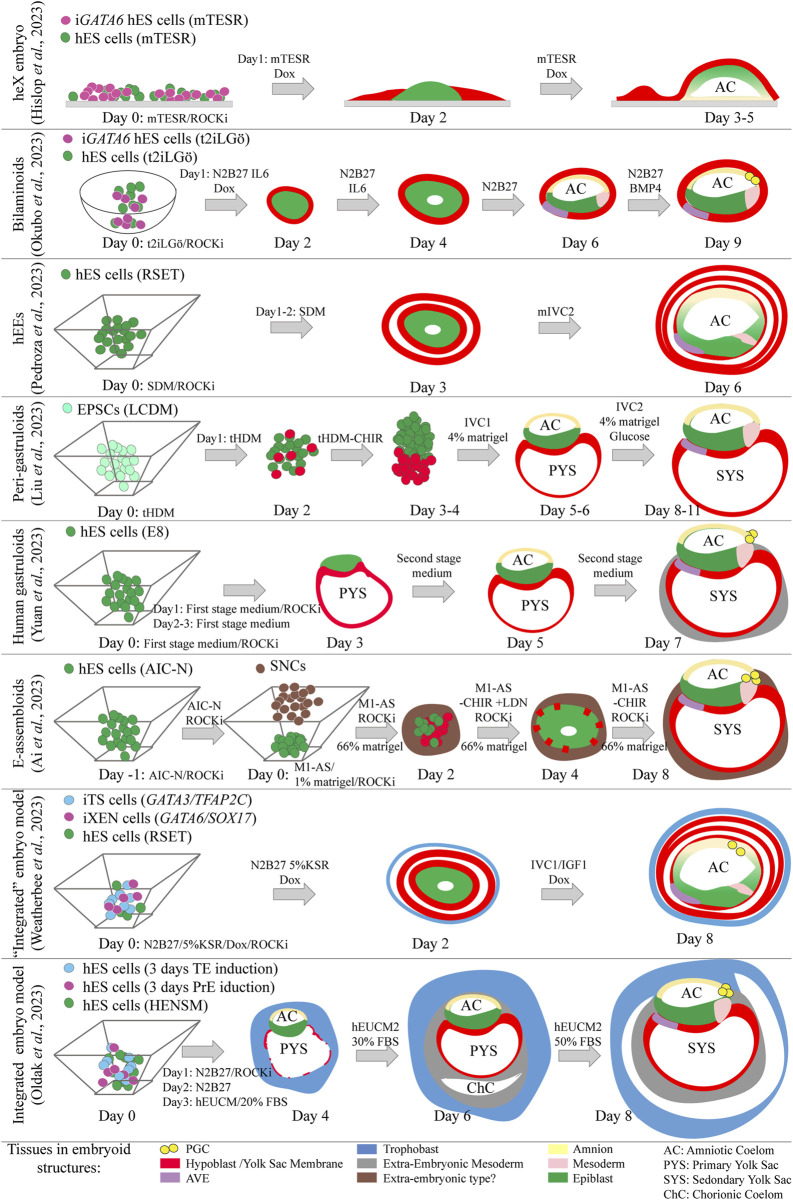
Schematic representation of recent partially and fully integrated stem cell-based embryo models mimicking early human post-implantation development. The upper six models do not display trophectoderm lineages, the bottom two models present epiblast, hypoblast and trophectoderm related lineages. Day 2 and day 4 representations of the E-assembloids are extrapolated from fluorescence images.

While extensive knowledge exists regarding mouse embryonic development, ethical constraints limit the study of human embryonic development. Nonetheless, valuable insights have been gained from the post-implantation amniotic sac embryoid (PASE) stem cell model (Shao et al., 2017a; Shao et al., 2017b). This model is a non-integrated stem cell-based embryo model that uses hES cells, and accurately simulates the development of the amniotic epithelium from the epiblast. It shows that the formation of the amniotic epithelium along PGCs, is dependent on BMP signaling (Zheng et al., 2019). The origin of BMP is speculated to be extra-embryonic. Subsequently, the amnion produces BMP4, triggering mesoderm formation in the posterior epiblast in a WNT-dependent manner (Shao et al., 2017a; Shao et al., 2017b; Zheng et al., 2019; Yang et al., 2021). However, there are still unanswered questions about the source of signaling molecules and the manner different lineages interact in these non-integrated stem cell-based embryo models that lack the extra-embryonic cell types. Human stem cell-based embryo models presenting extra-embryonic lineages could offer significant value in addressing these challenges. Six recent studies have presented stem cell-based embryo models featuring a bi-laminar disc structure comprising hypoblast and epiblast cells, while lacking a trophoblast compartment. The key commonality across all six studies is the induced formation of the hypoblast layer, facilitating the development of a bi-laminar disc structure. The models are known as heX embryoid, human extra-embryoid (hEE), peri-gastruloid, human gastruloid, E-assembloid and bilaminoid models (Ai et al., 2023; Hislop et al., 2023; Liu et al., 2023; Okubo et al., 2023; Pedroza et al., 2023; Yuan et al., 2023) (Figure 4). In heX embryoids (Hislop et al., 2023), hypoblast development relied on the continuous induction of *GATA6* transcription in primed hES cells. This induction occured while co-culturing hES cells expressing *GATA6* with non-induced hES cells on a cell culture dish. Consequently, induced cells encapsulated non-induced ones, leading to the formation of bi-laminar disc structures featuring an amniotic cavity. Notably, some hypoblast cells acquired characteristics similar to the AVE. Prolonged culture under modified conditions triggered the emergence of yolk sac mesoderm and blood progenitors (Hislop et al., 2023). In bilaminoids (Okubo et al., 2023), hypoblast induction relied on induced *GATA6* expression in naïve hES cells. Upon aggregation with naïve hES cells, the hES cells expressing *GATA6* encapsulated those that did not. Subsequently, the *GATA6* non-expressing ES cells gave rise to an amniotic cavity and epithelium, along with AVE-like, mesoderm-like, and PGC-like cells that were observable by day 9. The formation and expansion of the amniotic cavity relied on the addition of IL6 during the first 4 days, whereas the formation of PGCs and most likely also the amnion relied on addition of BMP4 from day 5 onwards (Okubo et al., 2023). The human extra-embryoids (hEEs) model employed hES cells, which underwent spontaneous differentiation toward hypoblast during the aggregation procedure in non-adhesive culture conditions (Pedroza et al., 2023). Human ES cells cultured in RSET (intermediate pluripotency) were chosen due to challenges in forming organized structures with both naïve and primed hES cells. This spontaneous differentiation led to a mixed cell population, with some cells biased toward hypoblast formation while others retained pluripotency. Co-culture with TS cells compromised structural organization, however in their absence organized structures could form. By day 6, the pluripotent cells had formed an amniotic cavity with cells resembling amnion, epiblast, and primitive streak. The hypoblast, predominantly present, encompassed the entire structure, with some cells exhibiting AVE-like characteristics (Pedroza et al., 2023). A fourth paper, outlining the peri-gastruloid model (Liu et al., 2023), used EPSCs as the cell source. During the first 4 days of aggregation in non-adherent culture wells, the cells or aggregates were exposed to culture media promoting hypoblast formation. A portion of the cells acquired the hypoblast fate, leading to their efficient organization into embryoids resembling post-implantation-like human embryos. By day 11, the peri-gastruloids had developed bilaminar discs, amniotic and yolk sac cavities, initiated gastrulation, formed PGCs, and even exhibited features of early organogenesis (Liu et al., 2023). In the fifth paper, primed hES cells were used to form post-implantation-like aggregates known as human gastruloids (Yuan et al., 2023). Upon exposure to culture media supporting nEnd formation (first stage medium), hES cells underwent differentiation into hypoblast-like cells, which subsequently organized into a hypoblast-like cell layer and formed a primary yolk sac. Subsequent exposure to BMP4 and bFGF in the second stage medium facilitated the formation of the amnion. Remarkably, by day 7, the human gastruloids exhibited anterior-posterior polarity, accompanied by the emergence of AVE-like cells, mesoderm, and PGCs (Yuan et al., 2023). Finally, embryo-like assembloids (E-assembloids) (Ai et al., 2023) were derived from naïve hES cells and partially differentiated hES cells, termed Signaling Nest Cells (SNLs), which transiently emerged during trophoblast induction and secrete WNT and BMP ligands. The naïve hES cells were aggregated 1 day prior to the addition of SNLs to the non-adherent wells. While SNLs themselves did not form the trophoblast compartment, their presence facilitated the efficient aggregation of cells into an organized structure resembling a bilaminar embryonic disc. By day 8, the E-assembloids presented an amniotic cavity and a yolk sac, both surrounded by an extra-embryonic cell type. PGC-like cells were detected and although not definitely confirmed, an anterior-posterior axis with cells resembling the AVE and mesoderm was identified. From these models; peri-gastruloids, human gastruloids, and E-assembloids exhibit remarkable similarity to human post-implantation embryos. The reduced dependence on intricate culture media, however, make models like peri-gastruloids of greater utility for studying lineage interactions.

Two additional recent studies reported on the development of embryoids capable of incorporating all three early lineages into embryo-like structures (Oldak et al., 2023; Weatherbee et al., 2023) (Figure 4). These models, similar to murine ETX embryoids, involve the assembly of three distinct cell types with the potential to form trophoblast, hypoblast, and epiblast. In one approach, a group used transgene-overexpressing hES cell lines to generate the extra-embryonic lineages (trophoblast and hypoblast) necessary for constructing the human post-implantation model (Weatherbee et al., 2023). This model shares similarities with the human extra-embryoids (hEEs) (Pedroza et al., 2023), albeit that the entire embryoid is surrounded by TE-like cells and PGC-like cells can be detected. However, it may not be classified as fully integrated. In the second approach, researchers successfully employed three lineage-biased stem cell lines to create a structure resembling a human post-implantation embryo (Oldak et al., 2023). They used extra-embryonic stem cell lines partially chemically transdifferentiated from naïve hES cells (Oldak et al., 2023). Morphologically, this study provided compelling evidence, including images demonstrating the similarity of the model to E13-E14 post-implantation human embryos. The efficiency of producing these models remains notably low, likely due to challenges in forming the extra-embryonic lineages.

Enhanced understanding of the embryonic and extra-embryonic early lineages within the human blastocyst is imperative for selecting appropriate cell lines and establishing precise culture conditions in the development of stem cell-based human post-implantation embryo models. Conversely, a diverse array of partially integrated human post-implantation stem cell models has demonstrated remarkable fidelity in modeling post-implantation development beyond gastrulation; using solely either EPSCs, naïve hES cells and even primed hES cells. This significant finding highlights both the plasticity of the human epiblast in efficiently forming the hypoblast and the crucial role of the epiblast in establishing its own signaling center—the amnion. The successful formation of partially integrated post-implantation models modeling embryonic development up to the gastrulation stage suggests that, depending on the research question, integration of the trophectoderm may not be necessary to model and study human early post-implantation development.

## Comparative analysis methods of stem cell-based embryo models to natural embryos

To accurately assess the fidelity of stem cell-based embryo models in replicating mouse or primate embryonic development, direct comparisons with natural embryos are indispensable. Since transcription factors are not lineage-specific, accurately localizing a subset of transcription factors within a morphologically similar model is insufficient for evaluating the similarity between the embryo model and the natural embryo. While single-cell RNA sequencing (scRNA-seq) of dissociated embryo models enhances cell type characterization, additional assessments using immunostaining to locate these cell populations within the model are indispensable. To facilitate these comparisons, it is essential to have access to scRNA-seq and immunostaining data from natural embryos. While these libraries exist for mouse pre- and post-implantation embryos (reviewed in (Posfai et al., 2021)), creating similar resources for humans poses ethical challenges. Given these ethical constraints, non-human primate embryos serve as a complement for investigating primate embryonic development. As such, valuable scRNA-seq and immunostaining data have been derived from human (Blakeley et al., 2015; Stirparo et al., 2018; Petropoulos et al., 2016; Yan et al., 2013) and non-human primate (Boroviak et al., 2018; Nakamura et al., 2016) preimplantation embryos, *in vitro*-cultured human (Zhou et al., 2019; Xiang et al., 2020; Mole et al., 2021; Ai et al., 2023) and non-human (Ma et al., 2019; Niu et al., 2019; Gong et al., 2023; Zhai et al., 2023) embryos into post-implantation stages, human terminated pregnancies (Tyser et al., 2021; Xu et al., 2023) and uterine-retrieved non-human primate embryos (Nakamura et al., 2016; Zhai et al., 2022).

The integration and comparison of data from multiple sources, such as transcriptomic profiles and immunostaining of natural embryos and stem cell-based models, however, is challenging. Transcriptomic data, while informative, may encounter discrepancies between experimental systems due to variations in protocols and employed techniques. Similarly, immunostaining of post-implantation embryos and their corresponding stem-cell based embryo models is complex, as it requires meticulous sample preparation, orientation, and sectioning. It is therefore important to consider technical nuances and potential sources of variability, when comparing natural embryos and stem cell-based embryo models. While dissociation-based approaches struggle to preserve tissue structure, thereby restricting expression analysis within the natural context, spatial transcriptomic technologies aim to overcome this limitation. Whereas some studies have employed spatial transcriptomics technologies to study mouse (Peng et al., 2019; Chen et al., 2022; Sampath Kumar et al., 2023; Srivatsan et al., 2021) and human (Xu et al., 2023; Zeng et al., 2023) embryogenesis, the employed techniques currently still face too many technical and computational challenges (reviewed by (Zhang et al., 2023; Zhou et al., 2023)) to be routinely used for comparative analyses.

## Discussion

Partially and fully integrated stem cell-based embryo models offer an innovative tool to study primate and mouse embryonic development. These models not only alleviate the need for sacrificing living pregnant mice but also avoid the use of human embryos for research purposes. Moreover, they offer a framework to explore scientific inquiries that are otherwise impossible or exceedingly challenging to study *in vivo*. Studying embryonic development using stem cell-based embryo models requires not only faithful replication of embryonic processes but also necessitates achieving a high level of efficiency in replicating these events. Indeed, given the complexity of biological responses, employing large sample sizes is essential for accurate quantitative analysis of results.

Human and mouse stem cell-based embryo models, whether partially or fully integrated, present distinct challenges. Firstly, developmental differences between mice and primates impact the approach to construct these models. For example, in mice, where the ExEc acts as a pivotal signaling hub directing gastrulation, the inclusion of a trophoblast compartment becomes imperative when modeling post-implantation development. In contrast, in humans, where the epiblast-derived amnion governs gastrulation, the inclusion of a trophoblast compartment in early post-implantation stem cell models is not as important. Variations in potency of existing stem cell lines in humans and mice also contribute to differences observed between mouse and human integrated stem cell models. Human stem cell lines, such as 8CLCs, EPSCs, naïve- and primed-pluripotent stem cells, demonstrate plenipotent characteristics and have been used as the primary cell source for forming human integrated stem cell-based pre- and post-implantation embryo models. In contrast, the development of these mouse models depends on the incorporation of multiple lineage-restricted stem cell populations. This results in two discernable strategies, the ‘inductive’ and ‘assembly’ approaches. While the inductive strategy uses complex culture media to direct lineage development, the assembly approach leverages the self-organizing potential of different stem cell lineages. Both strategies have their advantages and disadvantages. Assembly methods, such as those seen in models like ETX, require precise titration of cell numbers from various stem cell lines to accurately model the normal, dose-dependent effects of signaling factors during early embryogenesis. Meanwhile, the use of lineage-restricted stem cells, facilitates genetic perturbation studies in specific cell lineages. On the other hand, approaches based on ‘induction’ using plenipotent stem cells, as seen in human integrated post-implantation models, do not require as rigorous a titration of cell numbers but may not be as suitable for lineage-specific genetic perturbation studies, considering the uncertain developmental fate of a plenipotent cell. A deeper understanding of human early lineage specification may, in the future, aid in the development of integrated human embryo models constructed from lineage-restricted stem cell lines.

In addition to addressing biological challenges, it is imperative to consider technical and ethical limitations when developing models for studying post-implantation embryogenesis in mice and humans. Optimizing *in vitro* culture conditions with authentic embryos is important for refining the culture procedure and establishing a framework for comparing the models. However, due to the ethical limitations surrounding human embryo research, these 3D culture procedures may be more easily optimized using non-human primates. Additionally, non-human primates provide an opportunity to explore culture procedures beyond gastrulation and into organogenesis. The development of culture platforms that support placental development will most likely be pivotal to support development even further. Due to ethical restrictions that human integrated stem cell-based embryo models face (Hyun et al., 2020), the development of non-human primate integrated embryo models will also be instrumental. These non-human primate models offer an avenue to explore the developmental potential of these models beyond gastrulation and into organogenesis, both *in vivo* and during *in vitro* culture.

In conclusion, mammalian integrated stem cell-based embryo models have become instrumental tools for studying early embryogenesis in both mice and primates. The differences in embryonic development and stem cell biology between humans and mice have shaped and will continue to shape strategies used to construct these models.
